# Inverse and Concordant Mucosal Pathway Gene Expressions in Inflamed and Non-Inflamed Ulcerative Colitis Patients: Potential Relevance to Aetiology and Pathogenesis

**DOI:** 10.3390/ijms23136944

**Published:** 2022-06-22

**Authors:** Jan Söderman, Linda Berglind, Sven Almer

**Affiliations:** 1 Department of Biomedical and Clinical Sciences, Linköping University, 581 83 Linköping, Sweden; 2 Laboratory Medicine, Region Jönköping County, 551 85 Jönköping, Sweden; linda.berglind@rjl.se; 3 Department of Medicine, Solna, Karolinska Institutet, 171 77 Stockholm, Sweden; sven.almer@ki.se; 4 IBD Unit, Division of Gastroenterology, Karolinska University Hospital, 171 76 Stockholm, Sweden

**Keywords:** differentially expressed genes, gene set enrichment analysis, intestinal mucosa, RNA-seq, RNA sequencing, text analysis, transcriptome, ulcerative colitis

## Abstract

Ulcerative colitis (UC) arises from a complex interplay between host and environmental factors, but with a largely unsolved pathophysiology. The pathophysiology was outlined by RNA-sequencing of mucosal biopsies from non-inflamed and inflamed colon of UC patients (14 and 17, respectively), and from 27 patients without intestinal inflammation. Genes differentially expressed (DE), or present in enriched gene sets, were investigated using statistical text analysis of functional protein information. Compared with controls, inflamed and non-inflamed UC mucosa displayed 9360 and 52 DE genes, respectively. Seventy-three non-pseudogenes were DE relative to both gender and inflammation. Mitochondrial processes were downregulated in inflamed and upregulated in non-inflamed UC mucosa, whereas angiogenesis and endoplasmic reticulum (ER) stress were upregulated in both tissue states. Immune responses were upregulated in inflamed mucosa, whereas the non-inflamed UC mucosa presented both up- and downregulated gene sets. DE and enriched genes overlapped with genes present in inflammatory bowel disease genome-wide associated loci (*p* = 1.43 × 10^−18^), especially regarding immune responses, respiratory chain, angiogenesis, ER stress, and steroid hormone metabolism. Apart from confirming established pathophysiological mechanisms of immune cells, our study provides evidence for involvement of less described pathways (e.g., respiratory chain, ER stress, fatty-acid oxidation, steroid hormone metabolism and angiogenesis).

## 1. Introduction

Ulcerative colitis (UC) is one of two main subtypes (the other being Crohn’s disease) of inflammatory bowel disease (IBD) and constitute a chronic inflammatory disease of the colon [1,2]. The two main subtypes are both heterogeneous diseases with significant differences as well as similarities, and whose origin is predisposed by a complex interplay between genetic, microbial and environmental factors. Thus far, 241 risk loci have been associated with IBD via genome-wide association study (GWAS) approaches, and two thirds of these loci impart susceptibility to both diseases [3]. In addition, studies have examined the IBD transcriptome in the intestinal mucosa [4,5], in isolated epithelial cells [6] and in peripheral blood [7] and tried to define gene expression signatures for disease subtypes [8,9] and for the prediction of disease course and clinical response to drug treatment [4,10]. In spite of these advances, the pathophysiology of IBD is not fully explained, which justifies additional investigations and approaches to dissect the etiological and pathogenic factors involved.

Therefore, the aim of this study was to outline the pathophysiology of UC by analysing gene expression in mucosal biopsies obtained from both non-inflamed and inflamed colon of UC patients, and from patients without intestinal inflammation and not suffering from IBD. RNA-sequencing data were investigated for differentially expressed (DE) genes and for gene set enrichment. Statistical text analysis was applied in order to further elucidate the functional significance of proteins encoded by identified DE and enriched genes, and their etiological and pathogenic involvement in UC.

## 2. Material and Methods

### 2.1. Study Samples

Colorectal mucosal biopsy specimens were collected during routine endoscopies of adult patients investigated for a known IBD diagnosis or in the work-up for suspected gastrointestinal disorders (Table 1). Study biopsies were sampled from areas macroscopically assessed as either inflamed or non-inflamed and collected in parallel to and from the same areas as biopsies for histopathologic assessment. Each study biopsy was categorized as ‘inflamed’ or ‘non-inflamed’ based on a compound evaluation of the endoscopic findings as assessed by one experienced endoscopist (S.A.) and on a routine histopathologic assessment for inflammation. In total, fifty-eight colorectal biopsies were included for analysis by means of RNA-seq (1 transversum, 2 descendens, 53 sigmoideum and 2 rectum).

Twenty-nine UC patients were sampled for 17 biopsies from inflamed mucosa (UC.I) and 14 biopsies from non-inflamed mucosa (UC.nI). The inflamed UC biopsies were from the transverse colon (1), descending colon (1), sigmoid colon (13) and rectum (2), and the non-inflamed UC biopsies were from the descending colon (1) and sigmoid colon (13).

Patients without intestinal inflammation and not suffering from IBD were included as non-inflamed, non-IBD controls (Table 1; 27 patients). These patients were investigated by endoscopy for the purpose of colorectal cancer screening due to acromegaly (7 patients) or due to either anaemia or gastrointestinal symptoms such as abdominal pain, diarrhoea, constipation or blood in stool (20 patients). All control biopsies were sampled from the sigmoid colon and were assessed as non-inflamed mucosa in accordance with the histopathological analysis. No abnormal histopathological findings were observed along the investigated portion of the gastrointestinal tract for nineteen of these study subjects. The remaining eight study subjects had either celiac disease (1) or various pathological findings during endoscopy (1 diverticula, 2 polyps, 1 colorectal carcinoma, 1 radiation proctitis, 1 low grade adenoma, and 1 haemorrhoids).

The study was approved by the Regional Ethical Review Board in Linköping, Sweden (Dnr 2011/201-31 with amendment Dnr 2013/211-32). Written and informed consent was obtained from all participants.

### 2.2. Sample Preparation and RNA-Sequencing

Biopsy specimens were processed and RNA purified as previously described [11].

RNA-seq was carried out at the National Genomics Infrastructure node in Stockholm (Sweden). The concentration of the RNA samples were measured using a Qubit instrument and the Qubit RNA BR assay (Thermofisher Scientific, Waltham, MA, USA), and Agilent RNA Nano chip was used to assess the integrity of the samples (Agilent Technologies, Santa Clara, CA, USA). Libraries for sequencing were constructed using the TruSeq stranded total RNA kit with Ribo-Zero for depletion of ribosomal RNA (Illumina Inc., San Diego, CA, USA). Clustering was accomplished using the cBot system and samples were sequenced on HiSeq2500 with a 1 × 51 nucleotides setup using the HiSeq SBS Kit v4 chemistry (Illumina). Sequencing data were converted to the fastq format and demultiplexed using the bcl2fastq2 conversion software (Illumina Inc., San Diego, CA, USA).

### 2.3. Statistical Analysis and Bioinformatics

Unless otherwise specified, all data processing and statistical analysis were conducted within the R environment 3.6.0 (https://cran.r-project.org/, accessed on 26 April 2019), and all R packages used were obtained from either the Bioconductor project (https://bioconductor.org/, accessed on 26 April 2019) or the Comprehensive R Archive Network (https://cran.r-project.org/, accessed on 26 April 2019). The false discovery rate (FDR) was controlled for by adjusting all *p*-values for multiple testing using the Benjamini-Hochberg procedure. Adjusted *p*-values of ≤0.05 were considered significant.

### 2.4. Analysis of Differential Expression Using RNA-Seq Data

Reads were aligned to the primary GRCh38 assembly from Ensembl (http://ftp.ensembl.org/pub/release-85/fasta/homo_sapiens/dna/, accessed on 30 June 2017)) and mapped to genomic features (56,303 features corresponding to the official gene symbols provided by HGNC, https://www.genenames.org/ (accessed on 30 June 2017) using the R package Rsubread (version 2.0.1), with a median number of mapped sequencing reads of 14.0 million reads (5–95% range of 9.9–16.5 million reads).

For the RNA-seq count data (number of reads per gene) the R package DESeq2 (version 1.26.0) was used to estimate both library size factors for scaling and dispersion values for gene expression, fitting negative binomial generalized linear models, and significance testing the model coefficients using the Wald test. DE genes were identified by testing the coefficients against a log_2_-fold-change (log2FC) threshold of zero. A positive log2FC indicates an upregulated transcript level, whereas a negative log2FC indicates downregulation. Prior to running the DESeq2 functions, a minimal filtering was carried out to remove genes with low expression, by requiring at least 14 counts across all samples, which corresponded to at least one count per sample of the smallest sample group (non-inflamed colonic UC mucosa, *n* = 14) of the primary factor of interest, leaving 30,093 genes. Genes were further annotated with their official full gene names, Entrez gene IDs and chromosomal location using the R packages AnnotationDbi (version 1.48.0) and the org.Hs.eg.db (version 3.10.0). Genes without a full complement of annotations were removed, resulting in 21,965 genes for subsequent analyses. Individual factor variables (e.g., gender) or interactions (factor variables combined to a single variable, e.g., disease and inflammatory status) were either analysed by themselves or while controlling for an additional factor (e.g., combination factor disease and inflammatory status, controlled for gender).

For specific genes (e.g., genes that were differentially expressed in relation to gender), count values were extracted and transformed using the variance stabilizing transformation function of the DESeq2 package and analysed separately for differential expression in relation to inflammatory status using the two-sample Wilcoxon test.

### 2.5. Principal Component Analysis

Prior to principal components analysis (PCA), a variance stabilizing transformation was applied using the DESeq2 package, and the 500 genes with the highest variance were selected as the basis for PCA. Variance was estimated using the R package matrixStats version 0.56.0. PCA was performed using the prcomp function (stats package) and based on zero centred expression data scaled to unit variance.

Associations between factor variables and the first three principal components were significance tested using the two-sample Wilcoxon test.

### 2.6. Enrichment Analysis

A ranked list of genes (descending order) was computed by multiplying the direction (sign) of the fold change with the absolute value of the logarithm of the corresponding unadjusted *p*-value, thus generating a ranked list with up-regulated genes in the top and downregulated genes in the bottom.

Gene set enrichment analysis of Gene Ontology (GO; http://geneontology.org/, accessed on 30 June 2017) Biological Process (BP) terms was performed using ranked lists of genes and the R package clusterProfiler version 3.14.3 (5 × 10^7^ permutations, gene set size 5–500). Redundancy of enriched GO terms were compressed using the simplify function and a Wang similarity measure of 0.7. The remaining GO terms were further analysed and visualized using the Cytoscape (version 3.8.0) plugin ClueGO (version 2.5.7). Up- and downregulated GO terms were imported as separate lists and analysed together using a Kappa score of 0.2. Additionally, in order to avoid too many terms and an algorithm that does not converge on a solution, a more stringent setting was used for group comparisons that involved inflamed UC mucosa, i.e., the adjusted *p*-value cut-off was reduced to 0.01 and GO term fusion was applied to related terms.

The set of genes that were differentially expressed with respect to both gender and inflammation were examined by means of over-representation analysis, instead of gene set enrichment analysis, using clusterProfiler.

### 2.7. Text Analysis of UniProt Functional Information

DE genes and core enriched gene names were translated using the R package BiomaRt (version 2.42.1) to UniProtKB identifiers corresponding to manually reviewed Swiss-Prot records (http://www.uniprot.org, accessed on 26 November 2021). Functional information (i.e., UniProtKB column Function [CC]) were programmatically obtained with an R script employing functions of the httr (version 1.4.1) and readr (version 1.3.1) R packages.

Functional protein information was subjected to quantitative text analysis using the R packages quanteda version 3.0.0, quanteda.textstats version 0.94.1, quanteda.textplots version 0.94. Prior to text analysis the root form of inflected words were generated using the R package textstem version 0.1.4 in conjunction with the default dataset hash_lemmas of the lexicon package (version 1.2.1). Additionally, during text processing via the quanteda package, common words were discarded using both the smart list available in the R package stopword (version 2.2) together with a customized list of common words (e.g., act, however, isoform, mediate, play, resulting, target) to better harmonize with the UniProt functional information. Removed words were replaced by empty strings to preserve word adjacency and punctuations were retained. Multi-word expressions were identified and scored (Wald test z-statistics for the multi-word co-occurrence metrics) using a fixed length word combination of two (bigrams, i.e., word pairs). Furthermore, functional protein information was characterized by single word occurrences (unigrams), and assessed for differential group association (keyness), e.g., up- and downregulated genes. Keyness was assessed using the Chi-square test of independence, and with the Yates’s correction applied.

## 3. Results

### 3.1. Unsupervised Clustering Based on Gene Expression

Gene expression differences in relation to disease or not, to inflammatory status and to gender were evaluated along the first three principal components, which accounted for 62.8% of the gene expression variance among the samples (i.e., 52.9%, 5.6%, and 4.3%).

**Figure 1:**
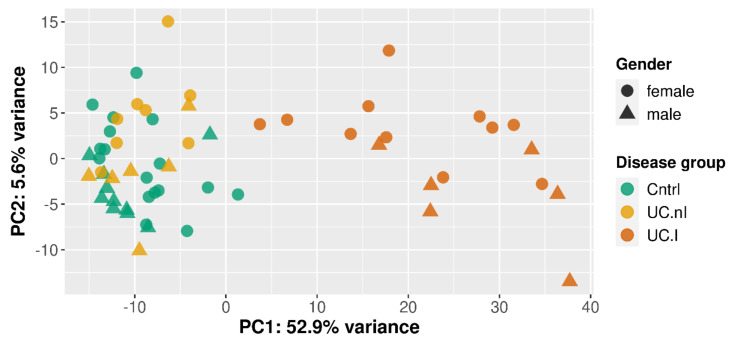
Visualization of disease groups and gender using the first (PC1) and second (PC2) principal component of the 500 genes with the highest variance. Disease groups were inflamed UC mucosa (UC.I), non-inflamed UC mucosa (UC.nI), and non-inflamed, non-IBD controls (Cntrl).

A separation in relation to presence or absence of inflammation was visually noticeable along the first principal component (Figure 1), so that inflamed UC mucosa (*n* = 17) clustered separately from non-inflamed mucosa from both controls (*n* = 27; *p* = 8.74 × 10^−12^) and UC patients (*n* = 14; *p* = 2.26 × 10^−8^).

Additionally, along the second principal component, control mucosa differed from both inflamed (*p* = 4.07 × 10^−2^) and non-inflamed UC mucosa (*p* = 2.68 × 10^−2^).

A gender associated difference in gene expression was observed in the second dimension for inflamed UC mucosa (6 males and 11 females; *p* = 5.82 × 10^−3^), but not for non-inflamed UC mucosa (6 males and 8 females) or control mucosa (10 males and 17 females).

Drug treatment involved five different drugs used either separately or in various combinations (Table 1), creating several small groups, which precluded a meaningful statistical evaluation in relation to principal components.

### 3.2. Differentially Expressed Genes

#### 3.2.1. Gender Based Differential Gene Expression and Inflammation

A stratified analysis of gender-based differential gene expression identified 48, 52 and 98 DE genes in non-inflamed control mucosa (10 males and 17 females), non-inflamed UC mucosa (6 males and 8 females) and inflamed UC mucosa (6 males and 11 females), respectively. Thirty-six of the DE genes were present in all three sample types, and in total, 120 DE genes (including 25 pseudogenes) were identified; 27 located on the Y chromosome, 22 on the X chromosome, and 71 with an autosomal location (Supplementary Table S1).

These genes were then separately analysed in relation to inflammation. Biopsies from males were evaluated for all 120 genes, whereas biopsies from females were evaluated for the 93 non-Y linked genes. In total 86 genes (including 13 pseudogenes) displayed differential expression in relation to inflammation (Supplementary Table S2).

When considering only the 73 non-pseudogenes, 37 genes were upregulated and 36 genes were downregulated with respect to inflammation. Out of the 37 upregulated genes, 26 genes showed a concordant regulation in males and females, three genes were upregulated only in females and eight only in males. Among males, the most upregulated genes (log2FC > 2) were *DMBT1*, *ALDOB*, *CXCL10*, *DSG3*, *SPP1*, *THBS2*, and *NPTX2*, whereas among females only *DMBT1*, *CXCL10*, and *STS* were upregulated at this level.

Out of the 36 downregulated genes, 19 genes showed a concordant regulation in males and females, four genes were downregulated only in females and thirteen only in males. Among males, the most downregulated genes (log2FC < −2) were *CLDN8*, *PRKG2*, *CAPN13*, *FAM189A1*, *ADGRV1*, and *MTRNR2L4*, whereas among females only *CLDN8* and *PRKG2* were downregulated at this level.

In general, these genes showed a more pronounced regulation with respect to inflammation in males compared to females, i.e., upregulated genes showed higher and downregulated genes lower log2FC values in males compared to females.

#### 3.2.2. Inflamed UC Mucosa vs. Controls

Inflamed mucosa from UC study subjects (UC.I; *n* = 17) was compared, while controlling for gender, to non-inflamed mucosa from controls (Cntrl; *n* = 27). This assessment resulted in 9360 DE genes (Supplementary Table S3); 4858 upregulated and 4502 downregulated in UC.I compared to Cntrl. Sixty-seven of the 73 non-pseudogenes that were identified as differentially expressed in relation to both gender and inflammation were also present among these genes. One hundred and eighty-seven of these genes showed an absolute log2FC of >3.32 (corresponding to a fold change of 10); 157 upregulated (adjusted *p* = 6.93 × 10^−63^–2.36 × 10^−4^) and 30 downregulated (adjusted *p* = 1.87 × 10^−27^–1.74 × 10^−5^).

Among the most upregulated genes were the regenerating family (e.g., *REG3A*), the serum amyloid A family of apolipoproteins (e.g., *SAA1*), the serpin family (e.g., *SERPINB7*), the matrix metallopeptidase family (e.g., *MMP7*), the solute carrier family (e.g., *SLC6A14*), the C-X-C motif chemokine receptor (e.g., *CXCR1*) and ligand (e.g., *CXCL8*, *CXCL1*, *CXCL11*, and *CXCL17*) family, the defensin alpha family (e.g., *DEFA5*), the S100 family (e.g., *S100A8* and *S100A12*), the claudin family (e.g., *CLDN2*, *CLDN14*, *CLDN18*), *AQP9*, *ABCA12*, *IL17A*, *ALDH1A2*, and *NOS2*.

Among the most downregulated genes were *G6PC*, *CYP3A4*, the solute carrier family (e.g., *SLC5A11*, *SLC6A4* and *SLC38A4*), *RIMS4*, *PDZK1*, *AQP8*, *CNTFR*, *PCK1*, *DIO3*, sulfotransferase family (e.g., *SULT2A1*), and *CLDN8*, and also genes that transcribed non-coding RNA (e.g., antisense RNA *C1QTNF1-AS1*).

#### 3.2.3. Non-Inflamed UC Mucosa vs. Controls

Biopsies from non-inflamed mucosa from UC (*n* = 14) and from controls (*n* = 27) were investigated for differential gene expression while controlling for gender. Out of 52 genes identified (including seven pseudogenes) as differentially expressed between these two groups (Supplementary Table S4), 41 were upregulated and 11 downregulated in UC.nI.

The most upregulated genes (log2FC > 2) among the 45 non-pseudogenes were *NPSR1*, *CCNO*, *CLDN2*, and *C2CD4B*.

None of the downregulated genes displayed a log2FC < −2. Three downregulated genes presented a log2FC < −0.62 (corresponding to a fold change of at least 1.53), i.e., *PEG10*, *KIT*, and *SEMA3D.*

### 3.3. Gene Set Analysis

Gene set enrichment analysis was employed in order to detect subtle and coordinated alterations in gene expression. To provide an overview, groups of GO BP terms (single instances or clusters of related terms) present in the ClueGO network were tentatively assigned to proposed biological themes. The assignments were supported by GO BP term definitions, co-occurring terms and associated child terms (https://www.ebi.ac.uk/QuickGO, accessed on 26 April 2019), and functional protein information based on manually reviewed Swiss-Prot records (http://www.uniprot.org, accessed on 26 April 2019).

#### 3.3.1. Gender and Inflammation DE Genes

An over-representation analysis of the 73 genes (excluding pseudogenes) with a differential expression in relation to both gender and inflammation revealed no significant GO BP terms. Nevertheless, several genes related to cell–cell adhesion, cell–extracellular matrix adhesion, barrier formation, epithelial cell proliferation and differentiation (e.g., *CFTR*, *CLDN8*, *CLDN23*, *CNTNAP2*, *DMBT1*, *DNMBP*, *DSG3*, *HAPLN1*, *IRF6*, *MFAP5*, *NRCAM*, *POF1B*, *PRKG2*, *SEMA3E*, *SPP1*, *TGFB3*, and *THBS2*), neurite outgrowth, axon guidance, neuroprotection, and neuronal reorganization (e.g., *CHN1*, *CNTNAP2*, *CXCL10*, *DRAXIN*, *MTRNR2L4*, *MTRNR2L10*, *NLGN4Y*, *NRCAM*, *PRKG2*, *SYT10*), and also to angiogenic and angiostatic factors (e.g., *CXCL10*, *CYP1B1*, *SEMA3E*, *THBS2*).

#### 3.3.2. Inflamed UC Mucosa vs. Controls

**Figure 2:**
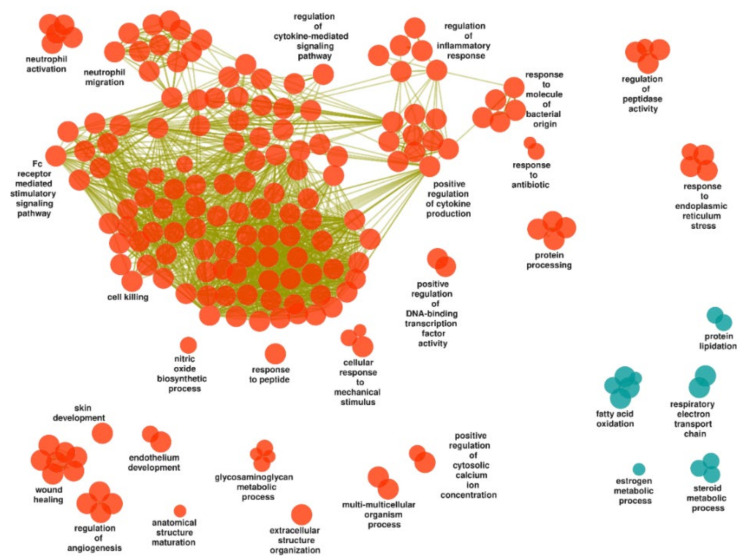
Gene ontology biological processes identified in inflamed UC mucosa compared to control mucosa and visualized as a functionally grouped network of gene sets that remain after ClueGO analysis. Orange and blue-green nodes represent upregulated and downregulated processes, respectively, and olive-green edges represent similarities based on gene content in each gene set.

Gene set enrichment analysis and simplification of GO BP terms resulted in 340 upregulated and 47 downregulated gene sets in inflamed mucosa of UC study subjects compared to controls (Supplementary Table S5). One hundred and eighty and thirteen terms, respectively, were retained by the ClueGO analysis, and visualized as a functionally grouped network (Supplementary Table S6 and Figure 2).

##### Upregulated Gene Sets

The vast majority of the upregulated gene sets concerned a tentative biological theme of host–microbe interactions, inflammation and immune response, and the largest group of terms (“cell killing”) encompassed processes such as: “T cell activation”, “B cell-mediated immunity”, “natural killer cell-mediated immunity”, “Fc receptor signaling pathway”, “defense response to bacterium”, “defense response to virus”, “complement activation”, “phagocytosis”, “response to interleukin-1”, “response to tumor necrosis factor”, “positive regulation of cell adhesion”, and “response to decreased oxygen levels”.

Additional groups related to the theme of host–microbe interactions, inflammation and immune response were “regulation of inflammatory response”, “positive regulation of cytokine production” and “regulation of cytokine-mediated signaling pathway”, “response to molecule of bacterial origin”, “neutrophil migration” and “neutrophil activation”, “Fc receptor mediated stimulatory signaling pathway”, “response to antibiotic”, “nitric oxide biosynthetic process”, “positive regulation of DNA-binding transcription factor activity” (with the term “positive regulation of NF-kappaB transcription factor activity”), “cellular response to mechanical stimulus”, “response to peptide”, and “regulation of peptidase activity”. Furthermore, embedded among these groups were “response to interferon-gamma”, “phagocytosis”, “complement activation”, “neuroinflammatory response”, and “response to decreased oxygen levels”.

Although not distinctly separated from the ongoing inflammation, other terms might be categorized as related to tissue homeostasis and remodelling, e.g., clusters and individual terms such as “wound healing”, “regulation of angiogenesis”, “endothelium development”, “anatomical structure maturation”, “extracellular structure organization”, “skin development”, and “multi-multicellular organism process”.

Further upregulated processes related to translation, modification and homeostasis of proteins, e.g., the clusters “response to endoplasmic reticulum stress”, “glycosaminoglycan metabolic process”, “protein processing”, and the previously mentioned “regulation of peptidase activity”.

##### Downregulated Gene Sets

Among the downregulated gene sets, terms related to cellular energy metabolism, i.e., “respiratory electron transport chain” and “fatty acid oxidation”, including the broader term “lipid oxidation”. Additional downregulated processes related to protein translation, modification and homeostasis (i.e., “protein lipidation”, including “GPI anchor metabolic process”) and to steroid metabolism (i.e., “steroid metabolic process” and “estrogen metabolic process”).

#### 3.3.3. Non-Inflamed UC Mucosa vs. Controls

**Figure 3:**
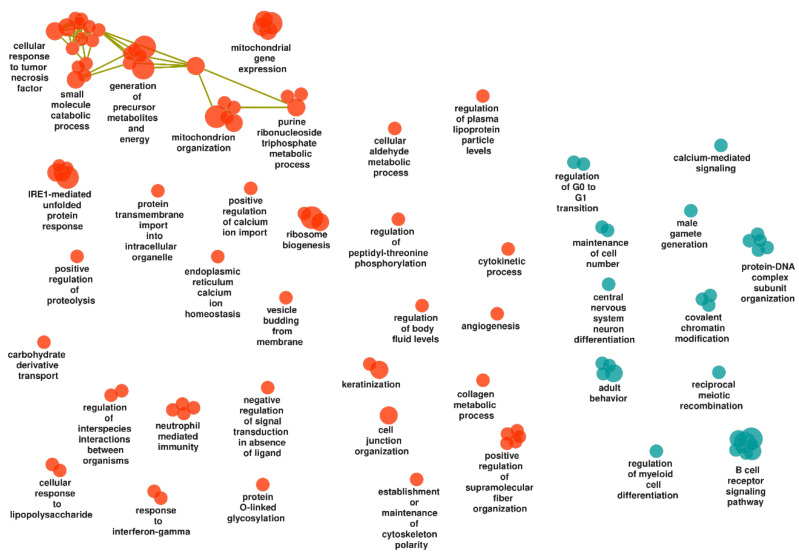
Gene ontology biological processes identified in non-inflamed UC mucosa compared to control mucosa and visualized as a functionally grouped network of gene sets that remain after ClueGO analysis. Orange and blue-green nodes represent upregulated and downregulated processes, respectively, and olive-green edges represent similarities based on gene content in each gene set.

Here we found 75 upregulated and 29 downregulated gene sets in non-inflamed mucosa of UC compared to that of controls (Supplementary Table S7). All terms were retained by the ClueGO analysis and visualized as a functionally grouped network (Supplementary Table S8 and Figure 3).

##### Upregulated Gene Sets

Among the tentative biological themes was host–microbe interactions, inflammation and immune response, e.g., clusters and individual terms such as “cellular response to lipopolysaccharide”, “carbohydrate derivative transport”, “regulation of interspecies interactions between organisms”, “neutrophil mediated immunity”, “response to interferon-gamma”, “small molecule catabolic process”, and “cellular response to tumor necrosis factor”. Both of the latter clusters included antigen processing via MHC class I, and the tumor necrosis factor response additionally related to hypoxia and to the epithelial cell layer.

Terms were also associated with tissue homeostasis and remodelling (e.g., “angiogenesis”, “cell junction organization”, “collagen metabolic process”, “keratinization”, “establishment or maintenance of cytoskeleton polarity”, “positive regulation of supramolecular fiber organization” (including “actin filament organization”) and “cytokinetic process”).

Furthermore, terms corresponding to cellular energy metabolism were among the upregulated gene sets of the non-inflamed UC mucosa, e.g., “mitochondrion organization”, “mitochondrial gene expression”, “purine ribonucleoside triphosphate metabolic process”, and “generation of precursor metabolites and energy” (involving the electron transport chain) and also “cellular aldehyde metabolic process”. The mitochondrial processes additionally involved transport, translation, and apoptosis. Moreover, this theme additionally related to the previously mentioned terms “carbohydrate derivative transport” and “small molecule catabolic process”.

Additional upregulated processes related to protein translation, modification and homeostasis, e.g., “ribosome biogenesis”, “IRE1-mediated unfolded protein response” (including “intrinsic apoptotic signaling pathway in response to endoplasmic reticulum stress”), and “positive regulation of proteolysis”.

##### Downregulated Gene Sets

The downregulated gene sets included the following tentative biological themes: host–microbe interactions, inflammation and immune response (e.g., “B cell receptor signaling pathway” and “regulation of myeloid cell differentiation”), cell population maintenance, and DNA and chromatin metabolism (e.g., “maintenance of cell number”, including “stem cell population maintenance”, “regulation of G0 to G1 transition”, “reciprocal meiotic recombination”, “male gamete generation”, “covalent chromatin modification”, and “protein–DNA complex subunit organization”), and neural cell differentiation, migration and neurotransmission (e.g., “central nervous system neuron differentiation” and “adult behavior”, including “regulation of neurotransmitter uptake”).

### 3.4. Core Enriched Genes and IBD Susceptibility Loci

Six hundred and seventy-one of the genes analysed in this study were located in known IBD GWAS loci [3]. Four hundred and thirty-three (64.5%) of these GWAS-related genes were identified as DE or core enriched genes in non-inflamed (85 up- and 51 downregulated genes) and inflamed (238 up- and 140 downregulated genes) mucosa of UC study subjects, resulting in a significant overlap (*p* = 1.43 × 10^−18^). In the inflamed UC mucosa, 198 of the GWAS-related genes were exclusively identified among DE genes (88 up- and 110 downregulated genes). Therefore, to further examine for enriched biological themes, quantitative text analysis (keyness, unigrams and bigrams) was applied to functional protein information associated with all identified DE and core enriched GWAS-related genes (Supplementary Table S9). As a group, these genes were most frequently associated with unigrams (Supplementary Table S10) describing regulation (e.g., receptor, signal, regulate, activation, response, induce, transcription, kinase, inhibit, cytokine, negative, phosphorylation, nf-kappa-b, histone, interferon, interleukin, tnf, and wnt), cell types, proliferation, migration and adhesion (e.g., t-cell, proliferation, differentiation, migration, macrophage, adhesion, b-cell, epithelial, dendritic, cell-cycle, endothelial, neuron, neutrophil, and nk-cell), and stress (e.g., degradation, stress, apoptosis, ubiquitin, ubiquitination, e3, survival, death, and chaperone). Both the endoplasmic reticulum and the mitochondria were among the unigrams (e.g., reticulum, endoplasmic, mitochondrial, and er), and lipid and fatty acid metabolism (e.g., lipid and fatty). These findings were further substantiated by bigrams (Supplementary Table S11).

#### 3.4.1. Inflamed UC Mucosa vs. Controls

In the inflamed mucosa, the largest number of upregulated GWAS-related genes were linked to ClueGO groups (Supplementary Table S12) of: (1) the immune system (e.g., cell killing, cytokine production and signaling, Fc receptor signaling, neutrophil activity, response to bacterial molecules, blood coagulation, and response to mechanical stimulus), with considerable overlap with gene sets relevant for (2) tissue remodelling and wound healing (e.g., angiogenesis, extracellular structure organization, nitric oxide biosynthetic process, skin development, anatomical structure maturation, and endothelium development), (3) protein metabolism (e.g., endoplasmic reticulum stress, protein processing of mainly components of the complement system, and glycosaminoglycan metabolic process), (4) regulation of cytosolic calcium levels, and (5) transcription factor activity.

**Figure 4:**
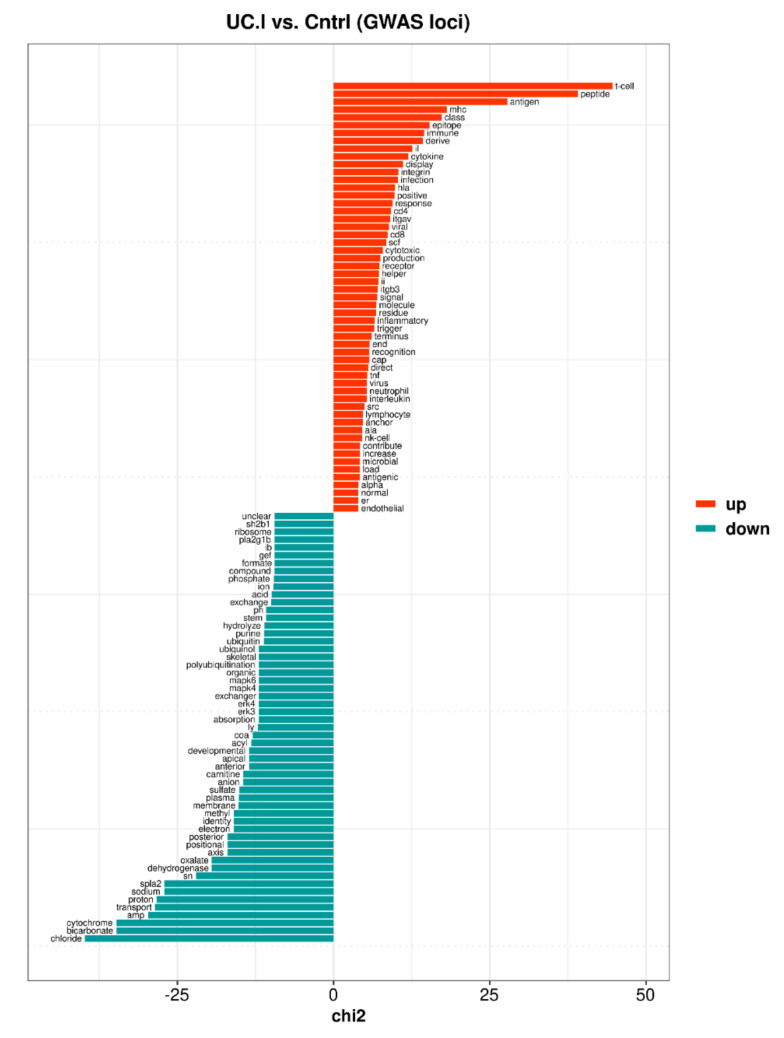
Top 55 keyness words describing a preferential association of unigrams with either upregulated (orange bars) or downregulated (blue-green bars) genome-wide association study (GWAS)-related genes of the inflamed UC mucosa (UC.I) compared to control mucosa (Cntrl). Keyness words were based on statistical text analysis of functional protein information (UniProtKB) associated with the GWAS-related genes. The chi2 value on the *x*-axis is the test statistic obtained from the Chi-square test used to calculate keyness.

Similarly, upregulated GWAS-related genes of the inflamed UC mucosa were preferentially described by keyness words (Supplementary Table S13 and Figure 4) associated with various aspects of: (1) the immune response (e.g., t-cell, mhc, cytokine, integrin, infection, cd4, viral, cd8, tnf, interleukin, neutrophil, nk-cell, microbial), (2) endothelial cells (i.e., endothelial) were commonly mentioned in an inflammatory context, affecting endothelial (dys)function, proliferation, migration, and interactions with leukocytes, and (3) the endoplasmic reticulum (ER) was in the context of MHC class II molecules, protein translocation into ER, protein transport through the Golgi apparatus to the cell surface, unfolded protein response, mitochondrial contacts, and autophagy.

Downregulated GWAS-related genes were linked to ClueGO groups corresponding to the respiratory electron transport chain, fatty acid oxidation, and steroid metabolism (Supplementary Table S12), and all were at least partly involved the mitochondria. Among the downregulated GWAS-related genes of the inflamed UC mucosa there were keyness words (Supplementary Table S13 and Figure 4) related to the mitochondrial respiratory chain and fatty acid-oxidation (e.g., cytochrome, proton, sodium, dehydrogenase, electron, carnitine, acyl, coa, ubiquinol, acid). However, keyness preferentially comprised words associated with an enrichment of genes involved in transport processes such as maintenance of pH and fluid homeostasis (e.g., chloride, bicarbonate, transport, proton, sodium, anion, absorption, exchanger, organic, pH, acid, ion). Furthermore, ubiquitin and polyubiquitination appeared among the keyness words, with relations to signaling (e.g., NF-kappa-B, MAPK, Wnt and CTNNB1), to cell proliferation, migration, and adhesion, and to the endoplasmic reticulum-associated degradation system.

#### 3.4.2. Non-Inflamed UC Mucosa vs. Controls

**Figure 5:**
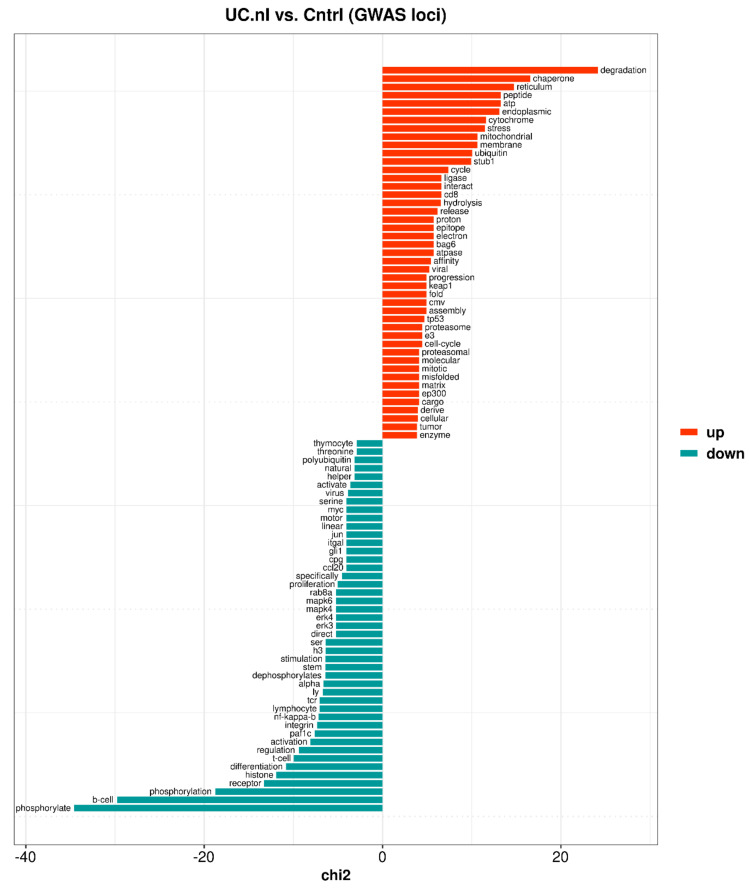
Top 45 keyness words describing a preferential association of unigrams with either upregulated (orange bars) or downregulated (blue-green bars) genome-wide association study (GWAS)-related genes of the non-inflamed UC mucosa (UC.nI) compared to control mucosa (Cntrl). Keyness words were based on statistical text analysis of functional protein information (UniProtKB) associated with the GWAS-related genes. The chi2 value on the *x*-axis is the test statistic obtained from the Chi-square test used to calculate keyness.

In the non-inflamed mucosa, the largest proportion of upregulated GWAS-related genes were found in ClueGO groups (Supplementary Table S14) associated with: (1) mitochondrial processes (e.g., energy production, organization, and gene expression), (2) immune responses (e.g., response to tumor necrosis factor, interferon-gamma and lipopolysaccharide, neutrophil mediated immunity, and interspecies interactions), (3) endoplasmic reticulum stress and protein metabolism (e.g., unfolded protein response, proteolysis, glycosylation, vesicle-mediated transport, and ribosome biogenesis), but also (4) angiogenesis, and (5) protein filament formation (e.g., keratinization, supramolecular fiber organization, and collagen metabolic process). These results were to a large part supported by keyness words associated with the upregulated GWAS-related genes (Supplementary Table S15 and Figure 5), e.g.: (1) cytochrome, mitochondrial, electron, proton, and matrix, (2) cd8, epitope, and viral, and (3) degradation, chaperone, endoplasmic, stress, ubiquitin, ligase, proteasome, and misfolded. However, keyness words corresponding to the ClueGO groups associated with angiogenesis and protein filament formation were not identified. Instead the text analysis identified regulation of cell cycle progression (e.g., progression, tp53, cell-cycle, mitotic). The keyness word atp was associated with both the energy generating mitochondrial respiratory chain and energy consuming processes such as protein folding and re-folding of misfolded proteins.

Downregulated GWAS-related genes were to a large extent associated with ClueGO groups (Supplementary Table S14) corresponding to: (1) immune cell regulation (e.g., mainly B cell receptor signaling, and also regulation of myeloid cell differentiation), and (2) chromatin modification and organization, and (3) to a lesser extent calcium-mediated signaling and (4) cell number and cell cycle regulation. Likewise, keyness words (Supplementary Table S15 and Figure 5) indicated a connection to the regulation (e.g., phosphorylate, regulation, activation, nf-kappa-b, stimulation) of immune responses (e.g., b-cell, receptor, differentiation, t-cell, integrin, tcr, proliferation), primarily involving T and B cells, but also to a lesser extent participation in histone modifications (e.g., histone, h3) and stem cell regulation (e.g., stem).

## 4. Discussion

Although the cause and driving mechanisms of UC largely remain unsolved, GWAS approaches and other findings have resulted in a widely accepted pathogenesis framework in which UC is attributed to a combination of genetic susceptibilities and intrinsic (i.e., the enteric microbial flora) as well as extrinsic environmental factors, involving an impaired integrity of the intestinal mucosal barrier, dysregulated innate and adaptive immune responses, and a dysbiotic commensal microbiome [3,12,13,14,15,16,17,18]. Moreover, experimental models of intestinal inflammation have contributed both to our understanding of pathogenetic mechanisms involved in IBD and to the development of drugs for the treatment of IBD [19].

In this study, we exposed several affected non-immune biological functions (i.e., functions of other cell types or ubiquitous cellular functions) alongside processes associated with innate and adaptive immune responses, both in inflamed and non-inflamed UC mucosa compared to control mucosa. We noted an inverse regulation of mitochondrial processes involving, e.g., a downregulation of the respiratory electron transport chain in the inflamed UC mucosa, and an upregulation in non-inflamed UC mucosa.

Although 241 IBD risk loci have been identified [3], out of which 193 have been defined as IBD-shared or UC-specific, this still only accounts for a small fraction of the variance in disease liability (i.e., less than 10%) [12,14], suggesting a considerable contribution from unidentified genetic variation, environmental factors or context-dependent interactions. Additional disease mechanisms have been described such as genetic susceptibility relating to endoplasmic reticulum (ER) stress and the unfolded protein response (UPR) [20]. Moreover, UC has been postulated as an energy-deficiency disease of the colonic mucosa [21], and more recent studies have described mitochondrial dysfunction as a potential disease mechanism [22,23].

### 4.1. Mitochondrial Dysfunction

Apart from a downregulation in the inflamed, and an upregulation in non-inflamed, colonic UC mucosa of components of the mitochondrial respiratory chain, we also noted this inverse regulation of gene sets relating to mitochondrial membrane organization, transport and gene expression, and that the non-inflamed mucosa additionally showed an upregulation of mitochondrial translation. A recent transcriptome study of inflamed rectal biopsies from a large cohort of paediatric UC patients described mitochondriopathy as a part of the pathogenesis, with a reduced gene expression affecting the citric acid cycle and the electron transport complexes I, III, IV and ATP synthase [4]. In addition, in the study, complex I and II of the electron transport chain were functionally assessed in control patients as well as in active and inactive areas of UC patients, using rectal and cecal biopsies, with a significantly decreased activity of complex I in mucosa from active UC. In a previous study of the four mitochondrial respiratory chain complexes, a reduced activity was detected in complex II, both in biopsies from abnormal areas of the rectosigmoideum and normal areas in the transverse or descending colon of UC patients compared to rectosigmoideal biopsies of control subjects [24].

In addition to the downregulation of mitochondrial functions, the inflamed UC mucosa revealed a downregulation of both fatty acid beta-oxidation and peroxisomal organization, transport, and protein localization. Fatty acid beta-oxidation takes place both in the mitochondrial and the peroxisomal compartment [25]. Carnitine is needed for the transfer of long-chain fatty acids as acylcarnitine esters into mitochondria for ATP production via beta-oxidation [25]. The carnitine transporter OCTN2 (*SLC22A5*), required for cellular uptake of carnitine, has been identified in a susceptibility locus for IBD [3], and the susceptibility variant has been associated with decreased levels of *SLC22A5* transcripts in Crohn’s disease [26]. In our study, *SLC22A5* was among the downregulated genes in inflamed UC mucosa (log2FC = −1.69, adjusted *p* = 5.30 × 10^−34^). Among the core enriched genes, we noted a downregulation in inflamed UC mucosa and an upregulation in non-inflamed UC mucosa of, for instance, *ACADS* (encoding a short-chain specific acyl-CoA dehydrogenase of the first step of mitochondrial fatty acid beta-oxidation), *SLC25A20* (encoding a transporter of acylcarnitines to the mitochondrial matrix) and *CRAT* (encoding a carnitine acyltransferase).

### 4.2. Endoplasmic Reticulum Stress

Processes relating to the ER, ER stress and proteasomal protein degradation were upregulated in both sample types. ER stress, due to the accumulation of unfolded or misfolded proteins, and the induction of the UPR (resulting in a resolution of ER stress or apoptosis) have been implicated in processes of potential relevance for IBD, such as differentiation, function and survival of immune cells (B cells, T cells, dendritic cells, and macrophages) and secretory functions and stemness of intestinal epithelial cells; both GWAS and candidate gene studies have identified genes with roles in relation to ER stress and UPR [27]. Resolution of ER stress include removal of misfolded proteins by proteasomal protein degradation. Studies on a mouse model of colitis support the relevance of colonic epithelial ER stress in the development of chronic colitis and implicate the interleukin-23/interleukin-22-axis as a driver of the ER stress response [28]. Furthermore, based on transcriptome data from colonic biopsies, this study also suggested the presence of a colonic epithelial ER stress in both UC patients and Crohn’s disease patients with colitis. Altered expression of ubiquitin-proteasome system components, in immune cells as well as intestinal epithelial cells, has been associated with IBD [29].

Compared to the findings of Powell et al. [28], the inflamed UC mucosa exhibited induced expression of *IL22*, *IL23A*, *IL12B* and *IL23R*, and also of genes of the interleukin-22 induced UPR ER stress module (*HSPA5*, *XBP1*, *ATF4*, *ATF6* and *HSP90B*). Furthermore, *HSPA5* was present among the core enriched genes of non-inflamed UC mucosa.

Notably, among individual genes relevant for UPR and ER stress, we identified *SDF2L1* (an IBD GWAS related gene that was DE in both inflamed and non-inflamed UC mucosa), *UBE2J2* (an IBD GWAS related gene that was DE in inflamed UC mucosa and core enriched in non-inflamed UC mucosa), and *YIF1A* (which was DE in both inflamed and non-inflamed UC mucosa).

### 4.3. Steroid Hormone Metabolism

In our study, inflamed UC mucosa showed evidence of ER-stress, reduced mitochondrial functions, and downregulated pathways for steroid metabolism. Lipids such as cholesterol are synthesized by the ER, and are impacted by ER-stress [30,31]. Steroids are formed from cholesterol imported to mitochondria via mitochondria-ER contact sites, lipid droplets or endosomes [32]. Both cholesterol and steroid metabolism have been implicated in intestinal inflammation. The *NR5A2* gene encodes a nuclear receptor implicated in processes such as cholesterol and bile acid homeostasis, steroidogenesis, glucose metabolism and crypt cell proliferation, and is found in a genome-wide associated region for UC [3]. Moreover, an impaired extra-adrenal glucocorticoid synthesis might contribute to the pathogenesis of intestinal inflammation [33], and genes encoding steroid metabolizing enzymes exhibit altered expression in inflamed colonic mucosa of IBD patients [34], i.e., an upregulation of *HSD11B1* (reactivation of cortisol) and a downregulation of *HSD11B2* (inactivation of cortisol). Further, a reduced expression of both *NR5A2* and steroidogenic enzymes as well as a reduced local glucocorticoid release have been demonstrated in experimental murine colitis and in biopsies from inflamed colonic mucosa of Crohn’s disease and UC patients [35], and in isolated colonic epithelial cells from UC patients [36]. Protective effects of *NR5A2* have been demonstrated with respect to the epithelial barrier and tumor necrosis factor-induced cell death using human and mouse intestinal organoids [37]. Isolated colonic epithelial cells obtained from UC patients exhibited a general downregulation of steroidogenic enzymes [36]. A general downregulation of genes affecting steroid synthesis were noted in our study, e.g., a downregulation of *NR5A2*, *FDX1* (transfers electrons to CYP11A1, which processes cholesterol to pregnenolone, the precursor of most steroid hormones), *HSD11B2*, and both *HSD3B1* and *HSD3B2* (involved in the biosynthesis of all classes of steroid hormones). In line with Stegk et al. [34], *HSD11B1* was upregulated in inflamed colonic UC mucosa. Glucocorticoids upregulate *PPARG* expression [36], an anti-inflammatory factor expressed by colonic epithelial cells, and in line with reduced steroidogenesis, *PPARG* also displayed a reduced expression in inflamed UC biopsies in our study.

### 4.4. Angiogenesis

Angiogenesis was upregulated in both sample types. Neovascularization is a feature of IBD, and both angiogenesis with elevated levels of integrin αVβ3 (i.e., *ITGAV* and *ITGB3*, expressed by angiogenic endothelium) and inducible levels of angiogenic factors (vascular endothelial growth factor, fibroblast growth factor 2, and interleukin-8) have been demonstrated in inflamed, but not in non-inflamed, colonic mucosa from UC and Crohn’s disease patients [38]. A role for vascular endothelial growth factor A (encoded by *VEGFA*) is established in the pathogenesis of IBD [39], with an increased expression in inflamed mucosa of both UC and Crohn’s disease patients. *VEGFB* has been identified in a susceptibility locus for IBD [3]. Caveolin-1 (*CAV1*), a main component of cholesterol-rich caveolae domains of endothelial plasma membranes, has been suggested to mediate pathologic angiogenesis in a mouse model of colitis [40].

All of these factors were significantly upregulated in the inflamed UC mucosa. However, among the vascular endothelial growth factor encoding genes, *VEGFC* was the most significant finding, whereas *VEGFB* was not differentially regulated. In this context, it is of special interest to note that among the angiogenesis core enriched genes that overlapped with IBD GWAS genes, *CXCR2* was the most upregulated gene of the inflamed UC mucosa. *CXCR2* mediates the angiogenic effects of interleukin-8 in intestinal microvascular endothelial cells [41]. Moreover, *CCL11* was among the top core enriched genes with respect to angiogenesis in the non-inflamed UC mucosa, with a borderline significant upregulation (FDR; *p* = 0.059), but significantly upregulated in the inflamed UC mucosa, and present in a IBD GWAS loci. *CCL11* induces angiogenic responses via its receptor CCR3 in endothelial cells [42], and the relevance of *CCL11* with respect to colitis has been demonstrated [43]. In addition, the *CCR3* gene is present among the IBD GWAS loci. Finally, expression of the angiogenesis-related *NOS3* gene (encoding the caveolae located endothelial nitric oxide synthase) was significantly upregulated in both non-inflamed and inflamed UC mucosa. Although the role of nitric oxide in IBD is unclear [44], a role for *NOS3* in the development of colitis has been demonstrated using murine models [45].

### 4.5. Intestinal Permeability

Tight junction genes of the claudin family were among the most differentially expressed genes in both inflamed (e.g., upregulation of *CLDN1* and *CLDN2*, and downregulation of *CLDN8*) and non-inflamed UC mucosa (e.g., upregulation of *CLDN2*), and were also present among genes associated both with gender and inflammation (e.g., downregulation of *CLDN8*). Mouse models with altered expression of specific claudin genes either develop spontaneous mucosal inflammation (*CLDN7* knockout) or exhibit enhanced (*CLDN1* overexpression, or *CLDN2* knockout) or reduced (*CLDN2* overexpression) susceptibility to induced colitis [46]. In line with the *CLDN7* knockout, we noted a slight but significant downregulation of *CLDN7*. Furthermore, in IBD patients that had achieved mucosal healing, but still experienced bowel symptoms, there were a correlation between bowel symptoms and an impaired intestinal permeability [47]. Although our previous candidate gene study identified a significant association between Crohn’s disease and a single nucleotide polymorphism in the region of *CLDN2*–*MORC4* [48], another study that investigated 128 barrier genes (including *CLDN2*) identified no single genes associated with either Crohn’s disease or UC [49]. Similarly to our present results, this latter study detected a large number of differentially expressed barrier genes, mainly in the inflamed mucosa, including tight junction genes.

### 4.6. Innate and Adaptive Immunity

A large number of innate and adaptive immune processes were markedly upregulated in the inflamed colonic UC mucosa compared to non-inflamed colonic mucosa from control subjects (e.g., both CD4 and CD8 T cell- as well as B cell-mediated immunity, antibacterial and antiviral defense response, cytokine production and signaling, NF-kappa-B signaling, and activities from neutrophils, macrophages, dendritic cells, natural killer cells, and the complement system). The immune response was not a major theme among the upregulated genes of the non-inflamed UC mucosa. Nevertheless, a few immune response-related genes were recognized based on Uniprot functional protein information, both genes with a positive (i.e., *C2CD4B*, *ISG20*, *CEACAM3* and the multifunctional *KIT*) and a negative influence (i.e., *SOCS3* and *NFKBIB*). Based on the gene set analysis, only a few upregulated immune processes (e.g., response to tumor necrosis factor, lipopolysaccharide, and interferon-γ, and neutrophil-mediated immunity) were present in non-inflamed UC mucosa, whereas other immune processes were downregulated (e.g., B cell receptor signaling and myeloid cell differentiation), thus demonstrating the non-inflamed state of the sampled area. It is feasible that residual differences between non-inflamed UC mucosa and non-inflamed colonic mucosa from controls represent baseline biological perturbations that predispose susceptible individuals for UC. The suitability of non-inflamed samples in the exploration of underlying mechanisms for disease initiation has also been suggested by another study identifying an upregulation of mitochondrial translation elongation and termination in non-inflamed UC mucosa compared to that of controls [50].

### 4.7. Differential Expression and IBD Susceptibility Loci

Upregulated DE genes and core enriched genes of the non-inflamed UC mucosa demonstrated an overlap with genes present in GWAS-loci that were associated with processes such as mitochondrial electron transport, unfolded protein response, proteasomal protein degradation, angiogenesis, interspecies interactions, and response to tumor necrosis factor, whereas downregulated GWAS-related genes were associated with processes affecting differentiation and activation of B cells. Likewise, upregulated GWAS-related genes of the inflamed UC mucosa were associated with T cell- and B cell-mediated responses, antigen presentation, cytokines, neutrophil activity, microbial infection, angiogenesis, and ER stress, whereas downregulated GWAS-related genes were associated with fatty acid beta-oxidation and carnitine transport, mitochondrial respiratory electron transport, and steroid metabolism.

Regulatory genetic variants can affect the expression of multiple genes, and within the IBD susceptibility loci there is enrichment of multiple coregulated genes that may be causally involved in disease predisposition [51]. Furthermore, investigations on healthy individuals suggest that regulatory variants, implicated in the majority of GWAS signals, act before disease onset [51]. In line with this, six proteins were upregulated in plasma samples obtained from individuals that developed UC later in life [52]. Three of these proteins were encoded by genes present among upregulated core enriched genes of the non-inflamed UC mucosa (*CCL2*, *CCL11* and *CXCL11*), and two of these (*CCL2* and *CCL11*) were present among the IBD GWAS-related genes. It is therefore conceivable that genes distinguished by the overlap between biological processes affected in the non-inflamed UC mucosa and genes in GWAS-loci associated with these processes are involved in a baseline susceptibility for developing active UC, whereas other GWAS-related genes and biological processes only manifest a perturbed expression once inflammation has been initiated.

## 5. Conclusions

In conclusion, by comparing distinctly separated inflamed and non-inflamed colonic biopsies from patients with ulcerative colitis and normal biopsies from controls without bowel inflammation, we were able to dissect several pathways of relevance to the aetiology and pathogeneses of UC. An extensive data set of mucosal gene expression was analysed and compared using several techniques including quantitative text analysis. Of pathophysiological pathways previously described in IBD, we identified and confirmed involvement of, e.g., the epithelial barrier function, and immune responses mediated by neutrophils, macrophages, T cells, and B cells. In addition, we found convincing evidence for the involvement of several less described pathways involved in the inflammatory cascades underlying IBD, mainly, mitochondrial function, endoplasmic reticulum stress, fatty-acid oxidation, steroid hormone metabolism, natural killer cells and angiogenesis, with all of them being promising for further characterization.

In a number of cases, we noted similarities between our results and results obtained using mouse models of colitis. However, UC has a complex aetiology and pathogenesis, and studies have demonstrated both similarities and differences between clinical disease and different mouse models of UC [53,54]. Thus, further studies with clinical samples and novel approaches are needed to clarify the aetiology and pathogenesis of UC.

## Figures and Tables

**Table 1:** Characteristics of patients with ulcerative colitis (*n* = 31) and non-inflamed controls (*n* = 27) ^a^.

	UC.I *n* = 17	UC.nI *n* = 14	Cntrl *n* = 27
**Gender** (male/female)	6/11	6/8	10/17
**Age** (years) ^b^	35 (18–68)	43.5 (23–65)	39 (19–81)
**Smoker** (yes/previous/no/no data)	3/3/9/2	0/1/12/1	0/2/19/6
**Concomitant drug treatment** ^c^			
AS	2	7	0
AS, CS	4	0	0
AP, TP	0	2	0
AS, CS, TP	2	1	0
ATA, CS, TP	1	0	0
CS	1	1	0
CS, TP	1	0	0
TP	0	1	0
None	6	2	27

^a^ Inflamed (UC.I) and non-inflamed (UC.nI) colorectal mucosa from UC patients were compared to non-inflamed colonic mucosa (Cntrl) obtained from a control group of patients referred for endoscopic examination due to gastrointestinal symptoms (e.g., diarrhea, fecal blood, or abdominal pain; *n* = 19), anemia (*n* = 1) or screening for colorectal cancer (*n* = 7) with the following findings: diverticulosis (*n* = 1), polyps (*n* = 2), low-grade dysplasia adenomas (*n* = 1), colorectal cancer (*n* = 1), hemorrhoids (*n* = 1), radiation proctitis (*n* = 1), or without any abnormal histopathological findings pathological findings (*n* = 20). ^b^ Median (range) values are given. ^c^ The following drugs were used individually or in combination: allopurinol (AP; 2 UC.nI), aminosalicylates (AS; 8 UC.I and 8 UC.nI), anti-TNF-α-antibodies (ATA; 1 UC.I), corticosteroids (CS; 9 UC.I and 2 UC.nI), thiopurines (TP; 4 UC.I and 4 UC.nI).

## Data Availability

The data presented in this study are available on request from the corresponding author. The data are not publicly available due to regulation regarding information that potentially could identify and compromise the privacy of research participants. Furthermore, the data constitute a part of our continued research, and therefore not publicly available.

## Supplementary Materials

The following supporting information can be downloaded at: www.mdpi.com/article/10.3390/ijms23136944/s1.

## Abbreviations

BP	biological process
Cntrl	controls
DE	differentially expressed
ER	endoplasmic reticulum
FDR	false discovery rate
GO	gene ontology
GWAS	genome-wide association study
IBD	inflammatory bowel disease
log2FC	log2-fold-change
PCA	principal components analysis
UC	ulcerative colitis
UC.I	inflamed UC mucosa
UC.nI	non-inflamed UC mucosa
